# Deep vein thrombosis and pulmonary embolism secondary to urinary retention: a case report

**DOI:** 10.1186/s13256-018-1605-3

**Published:** 2018-03-23

**Authors:** Tatsushi Kawada, Takashi Yoshioka, Motoo Araki, Hiroyuki Nose, Tadashi Oeda

**Affiliations:** 1 Department of Urology, Onomichi Municipal Hospital, 3-1170-177, Shin-Takayama, Onomichi-shi, Hiroshima, 722-8503 Japan; 2 Center for Innovative Research for Communities and Clinical Excellence (CiRC2LE), Fukushima Medical University, 1 Hikarigaoka, Fukushima-shi, Fukushima, 960-1295 Japan; 3 Department of Urology, Okayama University Graduate School of Medicine, Dentistry and Pharmaceutical Sciences, 2-5-1 Shikata-cho, Okayama-shi, Okayama, 700-8558 Japan

**Keywords:** Deep vein thrombosis, Pulmonary embolism, Urinary retention, Neurogenic bladder

## Abstract

**Background:**

Pulmonary embolism occurs when a blood thrombus forms and travels from a vein in the body to an artery in the lung. Thrombi often develop in one of the deep veins of the legs, thighs, or pelvis, a condition known as *deep vein thrombosis*. In this report, we describe a rare instance of a patient who developed deep vein thrombosis and pulmonary embolism secondary to urinary retention, and we also review some of the literature.

**Case presentation:**

A 75-year-old Japanese man visited our hospital with the complaint of lower extremity weakness. A physical examination revealed bilateral leg edema. Contrast-enhanced computed tomography showed thrombi in both the bilateral intrapelvic veins and the right pulmonary artery, with an extremely distended bladder. We diagnosed deep vein thrombosis and pulmonary embolism due to urinary retention, which was attributed to detrusor insufficiency owing to both taking an anticholinergic drug and neurogenic bladder. The patient was immediately started on both management of voiding dysfunction and anticoagulant therapy.

**Conclusions:**

We encountered a patient with deep vein thrombosis and pulmonary embolism secondary to urinary retention that could have been fatal. In such cases, clinicians should always take into account appropriate management of voiding dysfunction.

## Background

Pulmonary embolism (PE) induced by deep vein thrombosis (DVT) can be fatal; therefore, it should be considered when examining a patient with lower extremity edema. DVT is commonly seen in the lower limbs and pelvic veins. The common causes of thrombus formation are stasis of blood flow, endothelial injury, and hypercoagulability [1]. Stasis of blood flow is often caused by long-term bed rest, general anesthesia, lower limb paralysis, or varicose veins of the lower limb [2]. In this report, we describe a rare case of a patient with DVT and PE, which were caused by venous compression by an extremely distended bladder due to urinary retention. The characteristic computed tomographic (CT) image led to appropriate management.

## Case presentation

A 75-year-old Japanese man visited our hospital complaining of lower extremity weakness for a duration of 3 days. He had a past history of laminectomy for spinal canal stenosis and transurethral resection of the prostate for benign prostatic hyperplasia (BPH) and had been prescribed an anticholinergic agent, propiverine 20 mg/day, and a β3 adrenergic receptor agonist, mirabegron 50 mg/day, for treatment of urinary urgency by his family doctor.

**Fig. 1:**
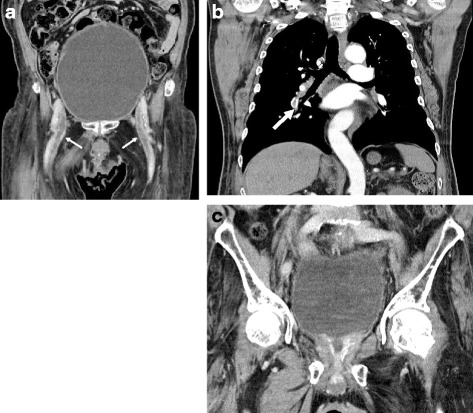
**a**, **b**; Contrast-enhanced computed tomographic scan shows thrombi in both the bilateral intrapelvic veins (small arrows) and the right pulmonary artery (large arrow), with an extremely distended bladder. **c**; Computed tomographic scan shows a postoperative change of transurethral resection of the prostate. The estimated prostate volume was 15 ml, which indicated that the patient did not have benign prostatic hyperplasia

A physical examination revealed bilateral leg edema. Laboratory examination showed that the patient’s d-dimer level was 7.7 μg/ml. Other laboratory test results were within normal limits. Chest radiography showed no sign of pleural effusion. Echocardiography showed no sign of left ventricular motor abnormality, but it revealed a mild hypertrophy of the left atrium that indicated increased right heart load. Contrast-enhanced computed tomography (CECT) showed thrombi in both the bilateral intrapelvic veins and the right pulmonary artery, with an extremely distended bladder. According to the CT scan, BPH was not present (Fig. 1).

**Fig. 2:**
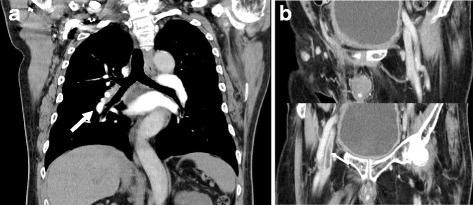
**a**, **b**; On the 15th day of the patient’s hospitalization, contrast-enhanced computed tomography showed that most thrombi (the bilateral intrapelvic veins; small arrow, the right pulmonary vein; large arrow) had resolved

We diagnosed DVT and PE due to urinary retention, which was attributed to detrusor insufficiency owing to both taking an anticholinergic drug and neurogenic bladder. The patient was hospitalized, a urethral catheter was inserted, and propiverine and mirabegron were discontinued. He was started on anticoagulant therapy with rivaroxaban 30 mg/day. On the second day of hospitalization, his lower extremity edema and lower limb muscle strength had improved bilaterally. On the ninth day, the urethral catheter was removed, and he was started on silodosin 8 mg/day and intermittent self-catheterization. On the 15th day, CECT showed that most of the thrombi had resolved (Fig. 2). On the 19th day, the patient was discharged. Anticoagulant therapy was maintained for 3 months, and the patient has reported no other events since the beginning of the treatment.

## Discussion

Our patient presented with DVT and PE secondary to urinary retention, which is a common symptom seen not only by urologists but also by general physicians and family practitioners. In the United States, DVT occurs in 50 per 100,000 people per year, and about 20% to 40% of DVTs are associated with PE [3]. A previous study identified mortality rates for PE of 4% at 1 month and 13% at 1 year [4]. Hence, DVT and PE require a rapid diagnosis and adequate treatment. Causes of DVT include venous stasis, endothelial injury, and hypercoagulability. DVT often occurs in the pelvic or lower limb veins and is commonly caused by increased venous pressure resulting from a congenital iliac band, iliac vein compression by the iliac artery, or an indwelling catheter from the thigh or long-term bed rest [2]. A site of predilection of DVT is the lower limb veins, and the initial site is commonly the soleus muscle vein [5]. In this case, thrombi were found in the femoral vein and external iliac vein, and no thrombi were observed below the popliteal vein. Imaging findings indicated that the cause of DVT was venous return failure caused by compression by an extremely distended bladder, in turn caused by urinary retention.

Urinary retention is a common urologic emergency in general practice [6]. Common causes of urinary retention are an outflow obstruction, a neurologic impairment, and an inefficient detrusor muscle [7]. The cause of urinary retention in this case was thought to be the patient’s inefficient detrusor muscle accompanying his taking an anticholinergic drug and a β3 adrenergic receptor agonist, as well as his neurogenic bladder accompanying lumbar spinal canal stenosis, because he did not have BPH, which is a common cause of urinary retention [7]. Drug-induced urinary retention is frequently observed in clinical practice, but anticholinergic drugs and β3 adrenergic receptor agonists can promote difficult urination associated with neurogenic bladder, which can be a cause of chronic urinary retention [8].

Venous obstruction by a distended urinary bladder was first described in 1960 by Carlsson and Garsten [9]. Since then, there have been several case reports describing an enlarged bladder compressing vascular structures in the pelvis [10–12], but few reports of cases that resulted in DVT and PE. Most of these were caused by BPH [13–17], whereas cases caused by neurogenic bladder are rare. Ito *et al*. [18] reported that clomipramine, prescribed for severely depressed and immobilized patients, caused DVT and PE as a result of urinary retention resulting from neurogenic bladder. Our patient’s case carries a significant clinical implication, namely that DVT and PE secondary to urinary retention caused by detrusor insufficiency due to both taking an anticholinergic drug and neurogenic bladder can occur even in patients who are not immobilized. Therefore, both physicians and urologists should pay attention to appropriate management of urinary dysfunction and prevent patients from developing chronic urinary retention, which can induce fatal complications.

## Conclusions

We treated a rare case of a patient with DVT and PE due to urinary retention. Although urinary retention is a relatively common disease condition, it should be noted that inappropriate management of urinary dysfunction may lead to fatal complications.

## Abbreviations

BPH: Benign prostatic hyperplasia
CECT: Contrast-enhanced computed tomography
CT: Computed tomographic
DVT: Deep vein thrombosis
PE: Pulmonary embolism
